# Supplementary material for: A protocol for high-dose quadrivalent influenza vaccine effectiveness in the community and long-term care facilities using electronic health records
Source: PLoS One. 2025 May 9;20(5):e0314177. doi: 10.1371/journal.pone.0314177 (PMC12064189; doi:10.1371/journal.pone.0314177)
Supplement: S1 Table — (PDF) [file pone.0314177.s001.pdf]

S1 Table. Secondary outcomes of interest by type of outcome, category, morbidity and respective ICD-10 code

Outcome	Category	Morbidity	ICD-10
**Primary**	**Infections**	**Influenza, virus not identified**	**J11**
		Influenza with pneumonia, virus not identified	J11.0
		Influenza due to unidentified influenza virus with unspecified type of pneumonia	J11.00
		Influenza due to unidentified influenza virus with specified pneumonia	J11.08
		Influenza with other respiratory manifestations, virus not identified	J11.1
		Influenza due to unidentified influenza virus with gastrointestinal manifestations	J11.2
		Influenza with other manifestations, virus not identified	J11.8
		Influenza due to unidentified influenza virus with encephalopathy	J11.81
		Influenza due to unidentified influenza virus with myocarditis	J11.82
		Influenza due to unidentified influenza virus with otitis media	J11.83
		Influenza due to unidentified influenza virus with other manifestations	J11.89
**Secondary**	**Infections**	**Viral pneumonia**	**J12**
		Adenoviral pneumonia	J12.0
		Respiratory syncytial virus pneumonia	J12.1
		Parainfluenza virus pneumonia	J12.2
		Human metapneumovirus pneumonia	J12.3
		Other viral pneumonia	J12.8
		Viral pneumonia, unspecified	J12.9
		**Pneumonia of unknown aetiology**	**J18**
		Bronchopneumonia, unspecified organism	J18.0
		Lobar pneumonia, unspecified organism	J18.1
		Hypostatic pneumonia, unspecified organism	J18.2
		Other pneumonia, unspecified organism	J18.8

		Pneumonia, unspecified organism	J19.9
	**Cardiovascular diagnosis**	Acute myocardial infarction or acute coronary syndrome	I20-23, I24-25
		Heart failure	I50, I51
	**Respiratory diagnosis**	Emphysema	J43.9
		Chronic obstructive pulmonary disease	J44.9
		Asthma	J45
		Dyspnoea/respiratory abnormality	R06.0
		Respiratory abnormality	R06.9
		Shortness of breath	R06.02
		Tachypnoea	R06.82
		Other respiratory abnormalities	R06.00, R06.09, R06.3, R06.89
	**Infections**	Other acute lower respiratory infections	J20-J22
		Viral infection, unspecified	B34.9
		Bacterial infection, unspecified	A49.9
		Bronchitis	J40, 41
		Myocarditis	I40.9
